# Breast Radiation Exposure of 3D Digital Breast Tomosynthesis Compared to Full-Field Digital Mammography in a Clinical Follow-Up Setting

**DOI:** 10.3390/diagnostics12020456

**Published:** 2022-02-10

**Authors:** Marcel Opitz, Sebastian Zensen, Katharina Breuckmann, Denise Bos, Michael Forsting, Oliver Hoffmann, Martin Stuschke, Axel Wetter, Nika Guberina

**Affiliations:** 1 Institute of Diagnostic and Interventional Radiology and Neuroradiology, University Hospital Essen, 45147 Essen, Germany; katharina.breuckmann@uk-essen.de (K.B.); denise.bos@uk-essen.de (D.B.); michael.forsting@uk-essen.de (M.F.); axel.wetter@uk-essen.de (A.W.); nika.guberina@uk-essen.de (N.G.); 2 Department of Obstetrics and Gynecology, University Hospital Essen, 45147 Essen, Germany; oliver.hoffmann@uk-essen.de; 3 West German Cancer Center, Department of Radiotherapy, University Hospital Essen, 45147 Essen, Germany; martin.stuschke@uk-essen.de; 4 Department of Diagnostic and Interventional Radiology, Neuroradiology, Asklepios Klinikum Harburg, 21075 Hamburg, Germany

**Keywords:** radiation exposure, digital breast tomosynthesis, mammography

## Abstract

According to a position paper of the European Commission Initiative on Breast Cancer (ECIBC), DBT is close to being introduced in European breast cancer screening programmes. Our study aimed to examine radiation dose delivered by digital breast tomosynthesis (DBT) and digital mammography (FFDM) in comparison to sole FFDM in a clinical follow-up setting and in an identical patient cohort. Retrospectively, 768 breast examinations of 96 patients were included. Patients received both DBT and FFDM between May 2015 and July 2019: **(I)** FFDM in cranio-caudal (CC) and DBT in mediolateral oblique (MLO) view, as well as a **(II)** follow-up examination with FFDM in CC and MLO view. The mean glandular dose (MGD) was determined by the mammography system according to Dance’s model. The MGD (standard deviation (SD), interquartile range (IQR)) was distributed as follows: **(I)** (CC_FFDM_+MLO_DBT_) **(a)** left FFDM_CC_ 1.40 mGy (0.36 mGy, 1.13–1.59 mGy), left DBT_MLO_ 1.62 mGy (0.51 mGy, 1.27–1.82 mGy); **(b)** right FFDM_CC_ 1.36 mGy (0.34 mGy, 1.14–1.51 mGy), right DBT_MLO_ 1.59 mGy (0.52 mGy, 1.27–1.62 mGy). **(II)** (CC_FFDM_+MLO_FFDM_) **(a)** left FFDM_CC_ 1.35 mGy (0.35 mGy, 1.10–1.60 mGy), left FFDM_MLO_ 1.40 mGy (0.39 mGy, 1.12–1.59 mGy), **(b)** right FFDM_CC_ 1.35 mGy (0.33 mGy, 1.12–1.48 mGy), right FFDM_MLO_ 1.40 mGy (0.36 mGy, 1.14–1.58 mGy). MGD was significantly higher for DBT mlo views compared to FFDM (*p* < 0.001). Radiation dose was significantly higher for DBT in MLO views compared to FFDM. However, the MGD of DBT MLO lies below the national diagnostic reference level of 2 mGy for an FFDM view. Hence, our results support the use of either DBT or FFDM as suggested in the ECIBC’s Guidelines.

## 1. Introduction

Breast cancer remains one of the most common cancers in women worldwide. In recent decades, the survival rate has improved [1], owing not only to better treatment strategies, but also to mammography screening programmes established in the Western world for detection of early stages of breast cancer. According to the International Agency for Research on Cancer (IARC), randomized controlled trials showed that screening may reduce breast cancer mortality by up to 40% for women aged 50–69 years [2]. Nevertheless, the established reference standard for screening, conventional full-field digital mammography (FFDM), is assumed to fail in the detection of 15–30% of breast cancers [3]. Hence, there is an ongoing debate about the optimal design of screening programmes, particularly regarding the optimal screening method [2] with new emerging screening modalities such as digital breast tomosynthesis (DBT), magnetic resonance imaging, or whole breast ultrasound examinations. According to a position paper of the European Commission Initiative on Breast Cancer (ECIBC), DBT is close to being introduced in European breast cancer screening programmes [4]. In order to improve the quality of breast cancer screening, diagnosis and care across Europe, either FFDM or DBT is recommended for screening purposes [4]. However, according to the commission initiative, only one technique should be used, not both [4].

Compared to standard FFDM as a common screening method, DBT has shown considerable improvement in screening detection and diagnosis of breast cancer, particularly in women with high breast density [3,5,6]. In most of the published screening studies, DBT was used additionally to FFDM in both views (cranio-caudal CC and mediolateral oblique MLO) for a better diagnostic accuracy [7,8]. However, using both modalities together in this way inevitably doubles the breast dose. Therefore, a simultaneous screening practice may not be appropriate because of the potential risk arising from general screening programmes, which apply ionizing radiation in mainly healthy women [9,10]. A real screening alternative must be capable of completely and solely replacing the present reference standard of breast cancer screening. Hence, several strategies have been introduced to achieve this goal. Software solutions have been implemented which combine the reconstruction of a synthetic biplanar mammography from the DBT [11]. This does not only offer a familiar, biplanar imaging view in breast cancer screening, but combines the advantages of both imaging modalities: standard biplanar mammography and DBT. Nevertheless, synthetic 2D mammography still has lower image quality than FFDM, so even with the use of synthetic 2D views, individual micro-calcifications are not equally well visualized.

Most existing studies compared FFDM and DBT regarding lesion conspicuity and diagnostic confidence. Several discussed radiation exposure of both modalities, but with variable results [12,13,14]. Hence, the purpose of this study was to compare the radiation dose delivered by digital breast tomosynthesis (DBT) and digital mammography (FFDM) to standard mammography alone (FFDM) in an identical patient cohort with different breast densities (ACR a–d) [11], and in a clinical follow-up setting.

## 2. Materials and Methods

### 2.1. Study Population

At our institute, it is mainly women with a certain risk of breast cancer who receive mammography scans. Generally, patients first receive an FFDM in CC-view. Depending on the breast density, the patients then receive an FFDM or DBT (optimal ACR c and d) image in MLO-view. After the installation of the DBT at our institute (April 2015), the indication for FFDM or DBT was deliberate. Therefore, in the time between May 2015 and September 2019, a total of 96 female patients with different breast densities (ACR b–d) received both (I) bilateral combination of standard mammography with tomosynthesis (FFDM and DBT) and (II) standard mammography alone (FFDM) in two separate sessions. Altogether, 768 breast examinations were acquired: FFDM in CC- and DBT in MLO-view (96 left, 96 right), as well as FFDM in CC- and MLO-view (96 left, 96 right) (Table 1). Between both examinations, patients underwent no substantial changes of parenchyma, viz. surgical removal of the breast. The patient cohort comprised 96 female patients with a mean age of about 64.1 years (age range 39–83 years). A total of 69 patients with breast cancer received both follow-up examinations after breast conserving therapy (BCT; 35 right, 32 left, 2 bilateral). The remaining examinations were carried out in order to evaluate suspicious findings by patients with mastopathy (10 patients) or micro-calcification (5 patients), and as follow-up in high-risk patients with a family history of ovarian carcinoma (1 patient) or breast cancer (8 patients). One patient received the mammography before a living kidney donation, and two patients as a follow-up to fibroadenoma. The local ethics committee granted ethical approval for this study (19-9096-BO).

### 2.2. Imaging Equipment and Dose Measurements

All examinations were performed with the same commercially available mammography system (Senographe Essential, GE MEDICAL SYSTEMS, Chicago, IL, USA; ADS_56.14.16 and ADS_56.21.3, update December 2016, Chicago, IL, USA), capable of both FFDM and DBT acquisitions. To test system performance and stability over time, daily and monthly quality controls (QC) were performed, as well as maintenance visits. Our institutional QC relies on the European QC approach, which seeks to provide a system-independent protocol in FFDM as well as in DBT, and continuously pursues new technological innovations [15,16,17].

All FFDM images were acquired in the “standard” FFDM setup and the automatic exposure mode. Thereby, a low dose pre-shot allowed the system to optimize the exposure parameters automatically (tube voltage in kV and tube load in mAs). For all DBT images, the system switched to the DBT mode. By using a step-and-shoot technique, 9 projections from −12.5 to +12.5 degrees were acquired. Equivalent to FFDM, a pre-shot in the first position was used for automatic optimization of parameters. In almost all examinations, the rigid paddle was used for breast compression; only in some cases the flexible paddle was used instead. The system selects mainly Rhodium/Rhodium as target/filter material, and for small breasts Molybdenum/Rhodium.

All examination parameters (mean glandular dose (MGD), entrance surface exposure (ESE), tube voltage, tube load, and compressed breast thickness) were retrieved from the control system. The breast densities (ACR) and clinical information, such as prior therapy, were extracted out of the clinical report.

To date, three different methods are applicable for MGD estimation, each of which has its limitations: (a) the Dance method [18], (b) commercial software solutions, and (c) the Wu method [19].

**(a)**  Estimating *MGD* according to the Dance method relies on the formula
  (1) MGD=IAK×g×c×s
  where ***IAK*** stands for the incident air kerma (without backscatter), ***g*** is the conversion factor for a breast with a defined glandularity of 50% by weight, ***c*** is the correction factor for breast composition, and ***s*** is the correction factor for X-ray spectra different from Mo/Mo [20]. MGD according to the Dance method may be derived from the DICOM header.
**(b)**  Estimating *MGD* taking the composition of the breast into account derived from commercial software solutions such as Volpara Solutions (Wellington, New Zealand), relying on the Dance model [21].
**(c)**  Estimating *MGD* following the Wu method relies on the formula
  (2) $$AGD=X_{ESE}\times D_{g}N\;$$
  where ***X_ESE_*** stands for the entrance skin exposure and ***D_g_N*** for the normalized glandular dose per unit entrance skin exposure, which differs for various anode/filter combinations and glandularities [22,23].

We decided to determine *MGD* as assessed in our clinical routine for dose monitoring purposes, hence, with algorithm (a). Here, MGD is provided by the mammographic equipment of GE Healthcare, which may be derived from the DICOM header. The densest area of the breast was calculated by the Senographe Essential system from a pre-exposure image. This was performed by the automatic exposure control (AEC) and an attenuation-equivalent thickness computed from a calibrated model.

### 2.3. Qualitative Image Analysis

The examinations were exported as DICOM files to the clinical PACS. The images were examined in a random order at separate time points by two experienced consultant radiologists. Image quality for all exams (DBT and FFDM in MLO view for each side) was visually rated based on 5-point Likert-scale [24], according to the detectability of (1) parenchyma distortions, (2) breast lesions, and (3) micro-calcifications. Additionally, both modalities were presented in a random order (FFDM or DBT) and rated regarding the confidence for defining the classification system for mammography Breast Imaging Reporting And Data System (BI-RADS) published by the American College of Radiology (ACR) [11].

### 2.4. Statistical Analysis

Kolmogorov–Smirnov and Shapiro–Wilk tests were applied to determine normal distribution. Pearson correlation analysis was used to identify positive correlation between technical parameters and MGD. Wilcoxon signed-rank test was performed to examine the differences of MGD between the two modalities (FFDM and DBT) separately for CC and MLO views. Kruskal–Wallis test was performed to examine dependence of MGD on breast thickness and ACR b–d group. A *p*-value lower than 0.05 was considered statistically significant. The descriptive statistics and statistical analysis were performed with the Statistical Package for Social Sciences v. 26.0. (SPSS Inc., New York, NY, USA).

## 3. Results

**Figure 1:**
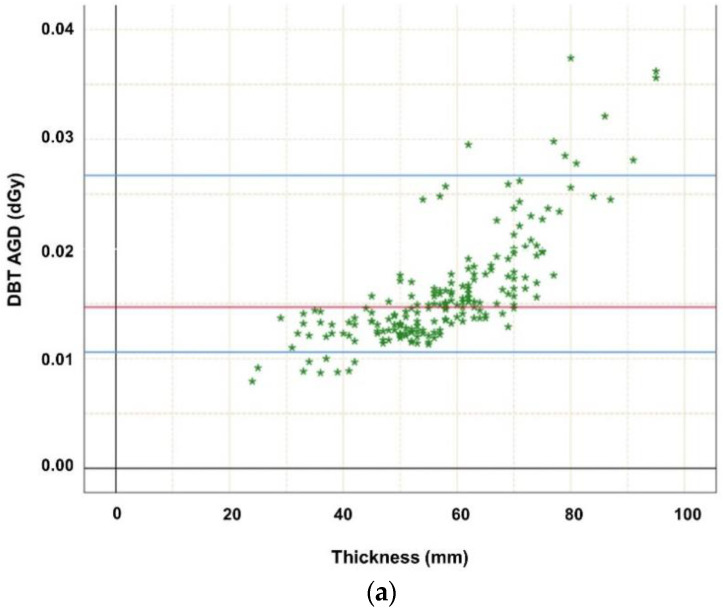

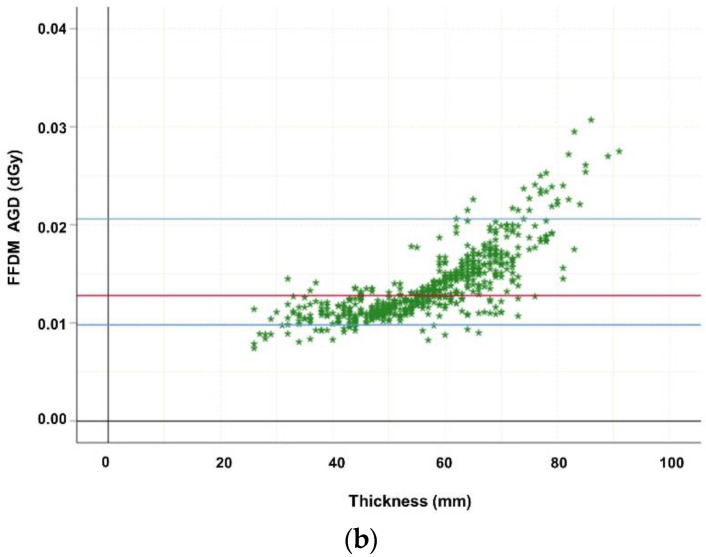
(**a**) Highlighting dependence of average glandular dose (in dGy) of digital breast tomosynthesis (DBT MLO projections; *n* = 192) on thickness. The MGD increase may be explained mainly by the automatic exposure control (AEC) which influences tube loading, kV, anode/filter combination, and breast composition such as thickness. Red line marks median MGD, the upper blue line marks the 95th percentile, while the lower blue line marks the 5th percentile. (**b**) Highlighting dependence of average glandular dose (in dGy) of digital mammography (FFDM cc and mlo projections; *n* = 576) on thickness. The MGD increase may be explained mainly by the automatic exposure control (AEC) which influences tube loading, kV, anode/ filter combination, and breast composition such as thickness. Red line marks median MGD, the upper blue line marks the 95th percentile, while the lower blue line marks the 5th percentile.

Descriptive statistics indicated that breast thickness was normally distributed, as assessed by the Kolmogorov–Smirnov and Shapiro–Wilk tests, *p* > 0.05. Pearson correlation analysis demonstrated a significant correlation of MGD of DBT with kV (*r* = 0.788, *p* < 0.001), mAs (*r* = 0.950, *p* < 0.001), exposure time (ms) (*r* = 0.965, *p* < 0.001), and breast thickness (*r* = 0.759, *p* < 0.001). The same is valid for MGD of FFDM with kV (*r* = 0.530, *p* < 0.001), mAs (*r* = 0.967, *p* < 0.001), exposure time (ms) (*r* = 0.860, *p* < 0.001), and breast thickness (*r* = 0.780, *p* < 0.001). The mean MGD (standard deviation (SD), interquartile range (IQR)) of both modalities was distributed as follows: (I) (CC_FFDM_+MLO_DBT_) (a) left FFDM_CC_ 1.40 mGy (0.36 mGy, 1.13–1.59 mGy), left DBT_MLO_ 1.62 mGy (0.51 mGy, 1.27–1.82 mGy); (b) right FFDM_CC_ 1.36 mGy (0.34 mGy, 1.14–1.51 mGy), right DBT_MLO_ 1.59 mGy (0.52 mGy, 1.27–1.62 mGy). (II) (CC_FFDM_+MLO_FFDM_) (a) left FFDM_CC_ 1.35 mGy (0.35 mGy, 1.10–1.60 mGy), left FFDM_MLO_ 1.40 mGy (0.39 mGy, 1.12–1.59 mGy), (b) right FFDM_CC_ 1.35 mGy (0.33 mGy, 1.12–1.48 mGy), right FFDM_MLO_ 1.40 mGy (0.36 mGy, 1.14–1.58 mGy). MGD was significantly higher for DBT MLO views compared to FFDM (*p* < 0.001) (Table 2). Statistical analysis showed statistically significant differences of MGD between FFDM and DBT for MLO views in this identical patient cohort (*p* < 0.001). Differentiating patient cohort according to breast thickness reveals that MGD was significantly correlated with breast thickness (Figure 1a,b, *p* < 0.001), which was only pseudo-linear, keeping in mind how automatic exposure control is set up.

**Figure 2:**
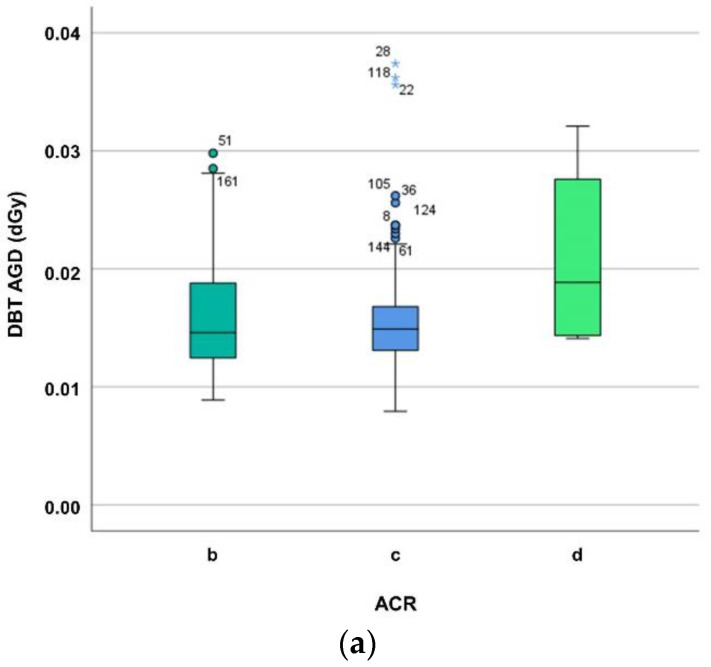

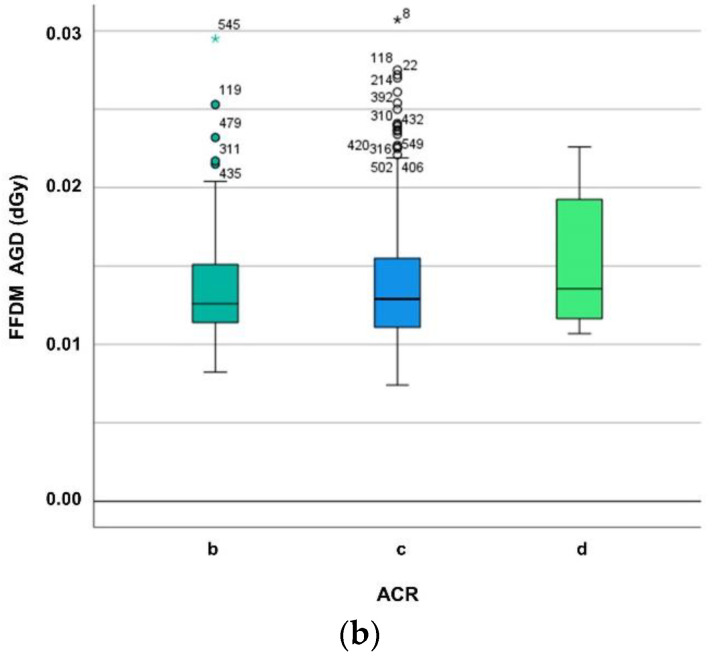
(**a**) Differentiating average glandular dose (in dGy) of digital breast tomosynthesis (DBT) for ACR breast density groups b–d. The MGD increase may be explained mainly by the automatic exposure control (AEC) which influences tube loading, kV, anode/filter combination, and breast composition such as breast density and thickness. (**b**) Differentiating average glandular dose (in dGy) of digital mammography (FFDM) for ACR breast density groups b–d. The MGD increase may be explained mainly by the automatic exposure control (AEC) which influences tube loading, kV, anode/filter combination, and breast composition such as breast density and thickness.

While Figure 2 delineates the dependence of MGD on breast composition, viz. ACR group, the Kruskal–Wallis test reveals no significant difference between ACR groups (for DBT *p* = 0.418, for FFDM *p* = 0.163).

Diagnostic confidence for tissue evaluation determined on a 5-pointed Likert scale is summarized in Table 3. Confidence for delineation of microcalcification in terms of absolute values was better in FFDM compared to DBT (rater 1 non-significant for both, left and right breast; rater 2 significant for both left and right breast *p* < 0.001), while delineation of parenchymal distortion, as well as of focal mass lesion, was superior in DBT compared to FFDM (rater 1 significant for both, left and right breast *p* < 0.001; non-significant rater 2, except for parenchymal distortions in the right breast).

## 4. Discussion

Regarding radiation exposure in medical imaging, the risk–benefit ratio is what matters and is of pervasive concern, particularly when examining radiosensitive tissue such as the female breast. This issue becomes even more evident in the setting of screening, where healthy women are examined in order to identify those who suffer from early stages of breast cancer. The present reference standard of breast imaging, mammography, proved to be a valid screening tool. However, there is an ongoing debate about the optimal design of screening programmes. An international panel of multidisciplinary members, the European Commission Initiative in Breast Cancer (ECIBC), recommends in its new guidelines that women be screened for breast cancer either with FFDM or DBT, especially in age groups 50–69 [4]. As prior studies concerning radiation exposure of these two modalities show variable results, the purpose of our study was to examine radiation exposure of FFDM and DBT in an identical patient cohort. The radiation risk of mammography is best represented by the mean glandular dose (MGD), because only the dose absorbed in glandular breast tissue is allied with a risk of cancer induction. Our results reveal that mean MGD is approximately 12% higher by DBT compared to FFDM in MLO view in patients with different breast densities (ACR b–d) at a comparable diagnostic confidence. These observations are valid for the entire cohort, as well as when differentiating in different ACR groups.

The present study results are in line with previous dosimetric examinations, which have shown that the MGD from DBT was slightly higher than from FFDM when using the automated exposure control mechanism by a system which is capable of both DBT and FFDM [13]. Bouwman et al. investigated MGDs for both dosimetric phantoms and for patients, for five different X-ray systems in FFDM and DBT. They found that the ratios between patient and phantom MGD did not considerably vary using two different dosimetry phantoms (polymethyl methacrylate (PMMA) or a combination of PMMA and polyethylene phantom) [16]. Depending on which of the two phantoms was used, the ratios in FFDM were 1.14–1.15 and in DBT 1.00–1.02 [16]. However, at the same time, they warranted that depending on various breast thicknesses, these ratios may differ substantially due to the automatic exposure control [16]. In general, the developments are going fast in DBT imaging direction. For the new types of systems of several manufacturers, radiation exposure of FFDM and DBT is now very similar.

Contrary to conventional mammography, where one biplanar X-ray image of the breast is obtained in MLO and CC projection, during a DBT, multiple low-dose radiographic images of the breast are acquired from various X-ray tube rotation degrees. During the multiple DBT projections, signal-to-noise ratio for each projection should be sufficient to overcome the multiple readout noise associated with the series of low dose images. For image reconstruction, DBT projections have to be acquired with a sufficient radiation dose, achieved by an appropriate tube voltage, tube current, and exposure time. According to Gennaro et al., automatic exposure control operates differently in mammography and tomosynthesis. While the MGD increase with mAs in FFDM is rather proportional, the MGD increase is faster in DBT because it is carried out using more penetrating spectra (heavier filtration/ higher kVp) [14]. However, to avoid excessive radiation dose by DBT, every DBT projection is acquired in a low dose mode. Furthermore, detectors with high dose efficiency are necessary [25]. Currently, there are two main DBT detector types applicable [25]: indirect conversion CsI:Tl scintillator coupled amorphous silicon detectors [26], and direct conversion amorphous selenium detectors [27]. In a review of radiation dose estimates, Svahn et al. examined DBT/FFDM MGD dose ratio and reported that a separate DBT was performed at lower to slightly higher radiation doses in comparison to FFDM [28]. While our results are rooted in an established clinical application setting, Svahn et al. report that most reviewed studies are almost entirely performed in an experimental or early clinical application setting [28]. Furthermore, in almost all reviewed studies, technical parameters were set manually rather than with the automated exposure control mechanism which is common practice in the clinical routine today [28]. Michell et al. compared conventional two view, film-screen mammography and two view, full-field digital mammography, reporting doses for conventional mammography of 1.37–1.57 mGy and for DBT of 1.66–1.90 mGy [29]. At our institute, for both modalities, but particularly for FFDM, MGD yielded lower levels. The authors in this study applied the dose-monitoring software tool Radimetrics Enterprise Platform^TM^ (Bayer Healthcare) discussed in Guberina et al. [30,31]. We examined radiation exposure of breast FFDM and DBT scans as implemented at our institute, strictly abiding by the recommendations of the vendor and image settings as determined by our expert breast radiologists for assessing clinical question of concern.

The main limitation of our study is the retrospective and single-centre design. Moreover, the obtained dose levels could differ from those obtained at other sites and mammography devices. With regard to the determination of the image quality of DBT and FFDM by two independent raters, it should be noted that the two raters have different levels of experience in observation and reporting of mammography examinations.

A major strength of our study is that all examinations were not only performed at the same mammography system, capable of both DBT and FFDM, but also on the identical patient collective. Breast tissue did not change during examinations, as patients received no medical treatment between both examinations, especially no surgical removal of the breast. This is of particular significance considering the fact that the automatic exposure control critically influences important technical parameters such as tube loading, voltage, and the anode/filter combination according to the individual imaged breast composition [28].

## 5. Conclusions

Our results confirm that MGD of DBT MLO lies within the limits of diagnostic reference levels published by the Mammography Quality Standards Act (MQSA) (3 mGy) [32] and below the Federal Office for Radiation Protection (BfS) for an FFDM (2 mGy) [33]. Moreover, DBT achieved confident to very confident marks for lesion detection and characterization, according to our diagnostic quality parameters. Hence, our results support the use of either DBT or FFDM in mammography screening, as suggested in the ECIBC’s Guidelines.

## Figures and Tables

**Table 1:** Number of images per each view and modality used in this study.

Examination	Modality	View	No. of Images
FFDM/DBT	FFDM	RCC	96
	FFDM	LCC	96
		Total CC	192
	DBT	RMLO	96
	DBT	LMLO	96
		Total MLO	192
FFDM	FFDM	RCC	96
	FFDM	LCC	96
		Total CC	192
	FFDM	RMLO	96
	FFDM	LMLO	96
		Total MLO	192

CC, cranio-caudal; DBT, digital breast tomosynthesis; FFDM, full-field digital mammography; MLO, medio-lateral oblique.

**Table 2:** Mean average glandular dose (MGD), standard deviation (SD), and interquartile range (IQR) per each view and modality.

Examination	Modality	View	Mean MGD (mGy)	SD (mGy)	IQR
FFDM/DBT	FFDM	RCC	1.36	0.34	1.14–1.51
	FFDM	LCC	1.40	0.36	1.13–1.59
	DBT	RMLO	1.59	0.52	1.27–1.62
	DBT	LMLO	1.62	0.51	1.27–1.82
FFDM	FFDM	RCC	1.35	0.33	1.12–1.48
	FFDM	LCC	1.35	0.35	1.10–1.60
	FFDM	RMLO	1.40	0.36	1.14–1.58
	FFDM	LMLO	1.40	0.39	1.12–1.59

MGD, mean glandular dose; CC, cranio-caudal; DBT, digital breast tomosynthesis; FFDM, full-field digital mammography; IQR, interquartile range; MLO, medio-lateral oblique; SD, standard deviation.

**Table 3:** Determination of image quality of digital breast tomosynthesis (DBT) and full-field digital mammography (FFDM) (mean; median values in parentheses; ***p***-values of Wilcoxon signed rank test analysing difference between DBT and FFDM) by two independent raters for the following features: (I) global confidence for BIRADS reporting; (II) parenchymal distortion; (III) focal mass lesion; (IV) microcalcification. Assessment based on a 5-pointed LIKERT scale: 5—extremely confident (for presence or absence of pathology); 4—very confident; 3—confident; 2—slightly confident; 1—not at all confident. Differentiation of left and right sided DBT and FFDM.

RATER 1	DBT		FFDM	
	Left	Right	Left	Right
Global confidence	4.19 (4.00) *p* < 0.001	4.17 (4.00) *p* < 0.001	3.58 (4.00) *p* < 0.001	3.53 (3.00) *p* < 0.001
Parenchymal distorsion	4.59 (5.00) *p* < 0.001	4.60 (5.00) *p* < 0.001	3.94 (4.00) *p* < 0.001	3.99 (4.00) *p* < 0.001
Focal mass lesion	4.17 (4.00) *p* < 0.001	4.20 (4.00) *p* < 0.001	3.37 (3.00) *p* < 0.001	3.38 (3.00) *p* < 0.001
Microcalcification	4.18 (4.00) *p* = 0.746	4.17 (4.00) *p* = 0.480	4.20 (4.00) *p* = 0.746	4.21 (4.00) *p* = 0.480
**RATER 2**	**DBT**		**FFDM**	
	**Left**	**Right**	**Left**	**Right**
Global confidence	4.55 (5.00) *p* = 0.238	4.42 (4.00) *p* = 0.501	4.46 (5.00) *p* = 0.238	4.47 (5.00) *p* = 0.501
Parenchymal distorsion	4.35 (4.00) *p* = 0.024	4.55 (5.00) *p* < 0.001	4.17 (4.00) *p* = 0.024	4.09 (4.00) *p* < 0.001
Focal mass lesion	4.24 (4.00) *p* = 0.893	4.29 (4.00) *p* = 0.065	4.23 (4.00) *p* = 0.893	4.15 (4.00) *p* = 0.065
Microcalcification	3.72 (4.00) *p* < 0.001	3.88 (4.00) *p* < 0.001	4.68 (5.00) *p* < 0.001	4.58 (5.00) *p* < 0.001
